# Practical Application of Passive Air-Coupled Ultrasonic Acoustic Sensors for Wheel Crack Detection

**DOI:** 10.3390/s25196126

**Published:** 2025-10-03

**Authors:** Aashish Shaju, Nikhil Kumar, Giovanni Mantovani, Steve Southward, Mehdi Ahmadian

**Affiliations:** Department of Mechanical Engineering, Virginia Tech, Blacksburg, VA 24061, USA; aashish00@vt.edu (A.S.); gmantovani@vt.edu (G.M.);

**Keywords:** railway wheel, crack detection, acoustic emission, ultrasonic sensing, non-contact monitoring, signal processing

## Abstract

**Highlights:**

**What are the main findings?**

- Passive air-coupled ultrasonic acoustic (UA) sensors were tested in both laboratory and track settings, where a reliable acoustic fingerprint for wheel damage was identified. The decay rate emerged as the primary diagnostic feature, achieving near-perfect ROC performance for severely damaged wheels.
- The acoustic fingerprint showed strong sensitivity to frictional and mass-loss defects such as shattered rim cracks and flange damage, but limited response to non-frictional surface conditions like Rolling Contact Fatigue (RCF), spalls, etc.

**What are the implications of the main findings?**

- This verified signature from UA sensors offers the “ground truth” needed to develop a practical, in-motion, passive acoustic monitoring system for railroad wheels, shifting the challenge from signal discovery to isolating targeted signals.
- The findings support a hybrid engineering approach for wayside deployment, combining physical acoustic focusing (e.g., waveguides) to improve signal-to-noise ratio with multi-feature machine learning models to enhance classification robustness.

**Abstract:**

Undetected cracks in railroad wheels pose significant safety and economic risks, while current inspection methods are limited by cost, coverage, or contact requirements. This study explores the use of passive, air-coupled ultrasonic acoustic (UA) sensors for detecting wheel damage on stationary or moving wheels. Two controlled datasets of wheelsets, one with clear damage and another with early, service-induced defects, were tested using hammer impacts. An automated system identified high-energy bursts and extracted features in both time and frequency domains, such as decay rate, spectral centroid, and entropy. The results demonstrate the effectiveness of UAE (ultrasonic acoustic emission) techniques through Kernel Density Estimation (KDE) visualization, hypothesis testing with effect sizes, and Receiver Operating Characteristic (ROC) analysis. The decay rate consistently proved to be the most effective discriminator, achieving near-perfect classification of severely damaged wheels and maintaining meaningful separation for early defects. Spectral features provided additional information but were less decisive. The frequency spectrum characteristics were effective across both axial and radial sensor orientations, with ultrasonic frequencies (20–80 kHz) offering higher spectral fidelity than sonic frequencies (1–20 kHz). This work establishes a validated “ground-truth” signature essential for developing a practical wayside detection system. The findings guide a targeted engineering approach to physically isolate this known signature from ambient noise and develop advanced models for reliable in-motion detection.

## 1. Introduction and Literature Review

### 1.1. The Critical Need for Railway Wheel Integrity Monitoring

The structural integrity of railroad wheels is essential for safe and efficient rail operations. Wheels endure extreme cyclic loading, thermal stresses, and environmental exposures that can cause gradual degradation. Railway wheels are subjected to extremely high contact pressures at the wheel–rail interface, making them vulnerable to defects that compromise the entire rail system [1]. If left undetected, these defects can grow into catastrophic failures, leading to derailments, operational disruptions, extensive damage to track and rolling stock, and major economic losses. For example, in North America alone, wheel-related failures have caused significant derailment incidents, with costs potentially reaching millions per event due to downtime, repairs, and liability [2,3]. Common defect types include subsurface-initiated shattered rim cracks, vertical split rims (VSRs), spalls, Rolling Contact Fatigue (RCF) networks, and flange cracks, all of which are identified in AAR/FRA programs and field manuals as condemnable or high-priority conditions. RCF [4], in particular, is a leading failure mode in heavy-haul freight operations, where repeated wheel-rail contacts generate shear stresses that initiate microcracks, eventually evolving into macroscale damage [5]. Additional studies and industry investigations show that shattered rims [6,7] usually begin below the tread at inclusions or voids and can spread quickly once they form, underscoring the importance of early, reliable detection.

Cracks in wheels can lead to failures and derailments, causing serious economic, social, and environmental damage. Economically, these incidents result in high costs from damaged tracks and trains, service delays, unexpected repairs, and legal claims. In North America alone, wheel and rail issues related to rolling contact fatigue (RCF) are estimated to cost more than 300 million dollars annually in replacements and repairs [4]. Socially, such derailments endanger human lives, potentially causing fatalities, injuries to crew and passengers, and wider community disruptions, while also eroding public confidence in rail safety and necessitating extensive emergency responses. Environmentally, they can create risks of hazardous material spills that contaminate soil, water, and air, leading to long-term ecological harm and cleanup costs. Broken wheel derailments, though rare, amplify these effects because they occur suddenly at high speeds, often resulting in more catastrophic outcomes compared to other failure modes [8].

### 1.2. State-of-the-Art in Wheel Inspection and Its Limitations

Traditional wheel inspections often use contact ultrasonic testing (UT), where probes with liquid or gel couplants are placed directly on the wheel to find cracks below the surface. This method is sensitive and reliable, but it is slow because it requires careful surface preparation and physical contact, making it difficult to apply during in-motion inspections. It also needs extra engineering efforts to keep the coupling consistent and to work at high inspection speeds [9]. To work around these problems, researchers have tested non-contact methods such as electromagnetic acoustic transducers (EMATs) and laser or air-coupled ultrasonics for wheels and rails. These approaches show promise, but they still face challenges with signal strength, background noise, and practical integration before they can be widely used [10,11,12].

Machine-vision portals equipped with high-speed, high-resolution imaging are widely adopted by the Class I railroads for automated surface inspection of rolling stock and wheelsets. However, these systems are fundamentally limited to detecting surface defects that are visible to cameras. Experience shows that the effectiveness of vision systems depends on factors like illumination, weather conditions, fouling or occlusion, and view geometry. Recent government technical assessments and reviews clearly identify lighting and weather sensitivity, occlusion, and target accessibility as factors that limit performance. They also emphasize that subsurface or early-stage cracks are beyond the detection capabilities of image-only methods [13,14,15].

Trackside microphones and acoustic emission (AE) sensors are used to monitor wheels and bearings, especially for detecting flats and other impact-related faults [16]. These systems work well for obvious surface issues but are less effective at detecting hidden cracks. Some studies using high-frequency AE show that faults in bearings and wheel-rail contact can be detected, but signals are often masked by other noises from trains and the environment. This highlights the need for better sensing methods and carefully chosen features to distinguish defect signals from background noise [17,18,19,20,21].

While promising, the above-mentioned non-contact methods such as EMATs and laser-air hybrid ultrasonics operate as active systems, requiring energy-intensive sources to transmit ultrasonic waves into the wheel and sophisticated receivers to analyze its reflection. This approach can achieve high detection resolution but often entails greater system complexity and cost. In contrast, the passive ultrasonic acoustic approach proposed in this study is fundamentally different. It is designed as a low-cost, low-complexity monitoring system that does not transmit energy but instead listens for the acoustic emissions naturally generated by defects during operational impacts. This distinction positions our work not as a direct replacement for high-fidelity active NDE, but as a complementary, highly scalable technology aimed at widespread screening and early-warning detection across the rail network.

### 1.3. The Proposed Passive Air-Coupled Ultrasonic Approach

To address these challenges, this project proposes the use of passive air-coupled ultrasonic acoustic emission (UAE) sensors for real-time crack detection as trains pass at operational speeds. The core concept treats cracks as “frictional energy concentrators” that emit broadband ultrasonic bursts during dynamic wheel-rail interactions, where interfacial friction at defect sites generates high-frequency acoustic energy that can be detected without external excitation. This approach offers significant advantages: it is passive, requiring no active probing; non-contact, using air-coupled sensors placed wayside outside safety clearances (e.g., satisfying the C-plate dimension); and suitable for in-motion deployment, enabling continuous monitoring during revenue service. By focusing on distinctive “acoustic fingerprints” from defects like shattered rim cracks, broken flanges, and RCF, the system supports proactive maintenance. This study aims to define and validate an acoustic fingerprint of wheel damage across ultrasonic (20–80 kHz) and sonic (1–20 kHz) bands and orientations, so that future wayside systems can identify and use this fingerprint for wheels in motion.

In acoustic emission-based railway monitoring, raw bursts must be processed through appropriate signal conditioning and feature extraction techniques in order to reveal useful diagnostic information. Prior studies have emphasized the importance of advanced signal processing models for isolating defect-related transients from background noise, such as the dynamic detection mechanism model of acoustic emission for high-speed train axle box bearings with local defects [22]. Building on this foundation, our work applies a structured pipeline for automated burst detection and feature extraction (described in Section 4), enabling quantitative comparison of diagnostic features across wheels with varying defect types and severities.

### 1.4. Research Challenges and Study Contributions

Early field tests identified a key issue: the overwhelming “wall of sound” from ambient wayside noise, characterized by broadband interference in the 20–80 kHz range that masks target crack signatures. This “reality gap” between controlled lab environments and noisy field conditions requires a structured approach to signal isolation. The main contribution of this study is to address this by shifting from searching for an unknown signal in a noisy environment to systematically defining, characterizing, and validating the ground-truth acoustic signature of wheel damage in a controlled setting.

Specific contributions include:

1. Development of a robust, automated pipeline for analyzing acoustic bursts.
2. Discovery and statistical validation of a new “acoustic fingerprint” primarily based on the decay rate feature.
3. Comparative analysis of the damage signature in both ultrasonic and sonic frequency bands was conducted to evaluate the feasibility of an alternative low-cost sonic sensor-based system. The results confirmed the hypothesized superiority of ultrasonics for passive wheel crack detection.
4. Validation of the acoustic fingerprint on a library of wheels with realistic, service-induced incipient defects.

This study is organized as follows: Section 2 summarizes the system setup and initial feasibility tests (Phase I); Section 3 discusses the wayside Signal to Noise Ratio (SNR) challenge; Section 4 describes materials, sensors, and the complete methodological framework; Section 5 presents the main results, from identifying the damage fingerprint to validating it on early-stage defects; and Section 6 explores the path toward in-motion deployment by addressing the key issue of signal isolation. Finally, Section 7 provides a summary of conclusions and outlines future work.

## 2. UAE System Setup and Initial Feasibility Tests

A high-fidelity, contactless measurement system was assembled to detect subtle, high-frequency acoustic emissions believed to originate from wheel defects. This section describes the system setup and presents initial laboratory tests to demonstrate its suitability for the intended purpose.

### 2.1. UAE Data Acquisition System Configuration

**Figure 1:**
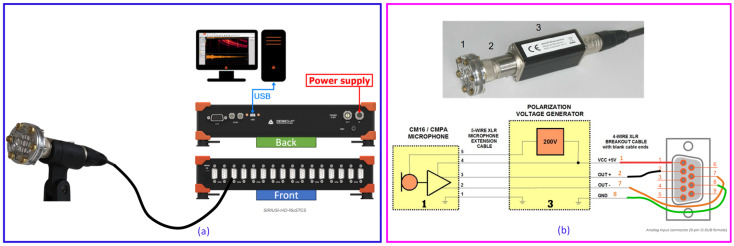
(**a**) Overall UAE measurement system configuration, showing the signal flow from the microphone to the SIRIUS DAQ and processing computer. (**b**) Detailed schematic of the Avisoft CM24 sensor system, showing the microphone capsule (1), preamplifier (2), and 200 V polarization voltage generator (3), along with the wiring diagram for signal and power to the DAQ’s analog input connector.

The data acquisition system, shown in Figure 1, was assembled to create a complete measurement chain from receiving acoustic waves to digital signal processing. The main sensing element is an Avisoft Bioacoustics CM24/CMPA condenser microphone (Avisoft Bioacoustics, Berlin, Germany). This sensor includes a CM24 capsule with a thin, metalized polyester diaphragm and a CMPA preamplifier module, making it highly sensitive to the broadband acoustic energy expected in the ultrasonic range (up to approximately 100 kHz).

As a condenser microphone, the CM24 capsule needs a stable, high-voltage bias to operate. This is supplied by an external polarization voltage generator, providing the required 200 V DC bias, as shown in the schematic in Figure 1b. The signal from the microphone’s preamplifier is routed through this generator, which then outputs the conditioned analog signal. Along with a +5 V DC supply voltage for the preamplifier electronics, this signal is connected via a breakout cable to a Dewesoft SIRIUSi-HD-16xSTGS data acquisition (DAQ) module (Dewesoft d.o.o., Trbovlje, Slovenia). The SIRIUS DAQ is a high-speed, multi-channel system capable of sampling simultaneously at 200 kHz per channel, ensuring high-resolution digitization of ultrasonic waveforms. This integrated configuration allows the DAQ to both power the sensor electronics and collect the resulting acoustic data, creating a self-contained and robust measurement loop suitable for both laboratory and field applications.

The selection of the ultrasonic band of interest (20–80 kHz) for this study was a deliberate decision based on a trade-off between diagnostic sensitivity and practical deployment constraints. While higher frequencies (>100 kHz) could theoretically offer greater resolution for micro-scale surface defects like RCF, their signals are subject to severe atmospheric attenuation. For a non-contact, wayside system, where sensors must be positioned at a standoff distance compliant with safety regulations (e.g., the FRA C-plate dimension), this potential signal loss could make reliable detection challenging. Therefore, we chose to first maximize the diagnostic information within the more robust 20–80 kHz ultrasonic band, providing sufficient spectral information for defect characterization while prioritizing robust signal propagation from the wheel to the sensor.

### 2.2. Laboratory Feasibility Validation

Before deploying a full-scale wayside system, a series of controlled laboratory experiments were carried out to confirm the basic physical principles of the proposed passive UAE detection method. The objective was to answer two fundamental questions: (1) Can crack-related acoustic emissions be generated by a controlled impact and detected by a non-contact sensor? and (2) Can the operational stresses of rolling contact alone generate a detectable passive signal?

#### 2.2.1. Static Impact and Dynamic Rig Evidence

**Figure 2:**
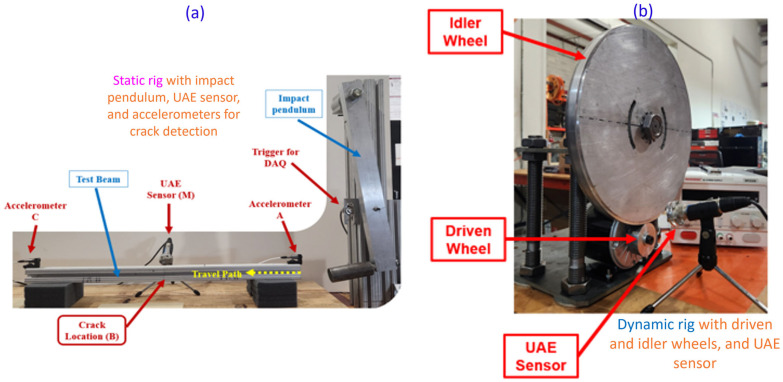
Laboratory setups for Phase I feasibility testing, showing the static impact rig with a test beam (**a**) and the dynamic rotating rig with an engineered-fault wheel (**b**).

**Figure 3:**
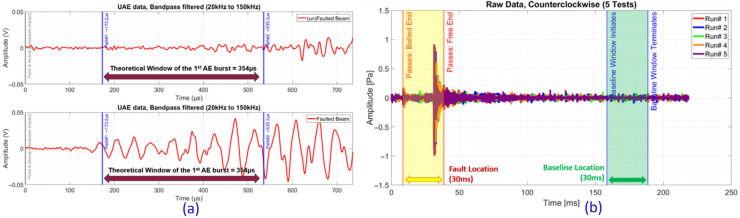
Positive UAE detection results from Phase I. (**a**) Filtered data from the static rig shows a clear difference in AE bursts between faulted and un-faulted beams. (**b**) Raw data from the dynamic rig shows repeatable, high-amplitude signal variations as the fault location passes the sensor, compared to the baseline.

To answer the first question, a static impact rig was used, as shown in Figure 2a. This setup included a test beam instrumented with a UAE sensor and accelerometers. Beams with and without engineered faults (i.e., manufactured cracks) were excited using a repeatable mechanical impulse from a pendulum. The results provided clear evidence of UAE detection; as shown in Figure 3a, a distinct, high-amplitude acoustic emission (AE) burst was consistently recorded from the faulted beam within the theoretically calculated arrival window, a signature that was absent in the un-faulted control beam.

Building on this success, a custom dynamic test rig was developed to validate the principle of purely passive detection (Figure 2b). This setup featured a loaded, rotating “idler wheel” with an engineered crack, driven by a second wheel to simulate the dynamic stresses of wheel-rail contact. This experiment was essential for determining whether operational stresses alone were sufficient to generate a detectable signal. As shown in Figure 3b, the UAE sensor successfully captured high-amplitude, repeatable signal bursts precisely as the fault location passed through the point of contact. These bursts were distinctly different from the lower-amplitude signals recorded from the unfaulted portions of the wheel, confirming that passive detection is viable.

#### 2.2.2. Key Insight

The key finding from this foundational phase was confirming that cracks serve as effective frictional energy concentrators within the wheel structure. Whether under direct impact or continuous stress from simulated rolling contact, frictional slip at the crack interfaces generated detectable, broadband ultrasonic emissions that could be captured by a non-contact, air-coupled sensor. These early laboratory results confirmed that the proposed passive UAE sensing method was physically viable, providing scientific confidence and justification to proceed with wayside field testing.

## 3. Initial Field Deployment and Wayside Acoustic Challenge

Transitioning from controlled laboratory validations in Phase I, the initial field test on revenue service tracks assessed the UAE system’s performance in a real-world environment. This deployment aimed to capture ultrasonic signatures from passing freight trains but quickly revealed the complexities of wayside acoustics, highlighting the “reality gap” between theory and practice.

### 3.1. Trackside Setup

**Figure 4:**
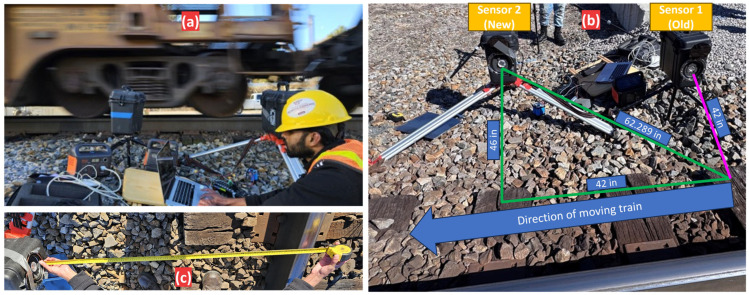
Field deployment of the UAE sensor suite. (**a**) Overall setup capturing data from a passing freight train. (**b**) Geometric arrangement of the two sensors relative to the track. (**c**) Measurement of the standoff distance from the railhead.

The field test was performed on an active revenue service track, monitoring freight trains traveling at speeds of 25–30 mph (40–48 km/h). As shown in Figure 4, the setup used two air-coupled ultrasonic microphones (UAE sensors) placed about 43 inches (1.09 m) away from the nearest railhead. This placement was selected to meet the C-plate safety dimension, ensuring a non-intrusive setup outside the train’s clearance envelope. Data from both sensors were acquired simultaneously at a sampling rate of 200 kHz using a ruggedized portable data collection system. This deployment successfully gathered a large amount of ultrasonic acoustic data from a variety of in-service railcars, marking the first real test of the UAE concept in a wayside environment.

### 3.2. The Wayside “Wall of Sound” and a New Problem Statement

Analysis of the field data revealed a key challenge that distinguished the outdoor environment from the controlled laboratory: the presence of a strong, non-stationary acoustic background. This environment was dominated by both continuous broadband mechanical noise and frequent, high-energy transient bursts originating from various sources, including draft gear slack action, suspension component movement, wheel-rail stick-slip, and impacts from rail joints.

**Figure 5:**
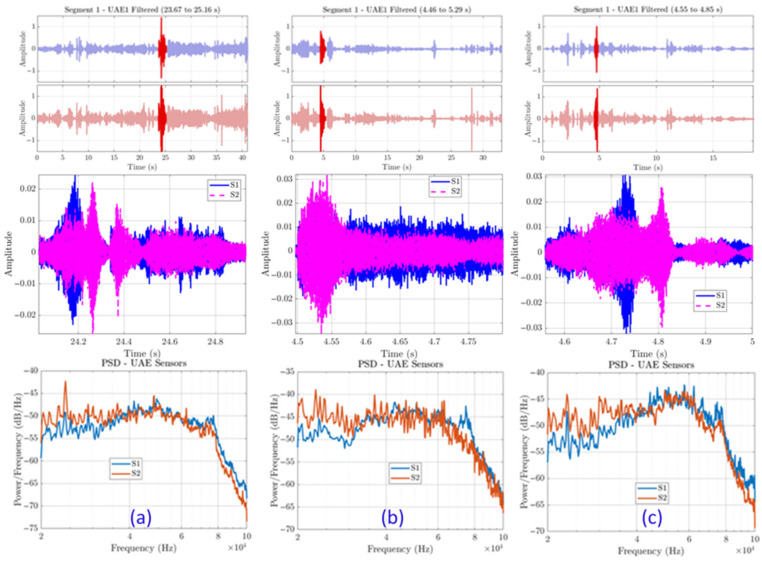
Analysis of three distinct data segments from the field test. For each event (**a**–**c**), the time-domain signal and the corresponding PSD plot show a consistent pattern of high-amplitude, broadband noise in the ultrasonic frequency range.

**Figure 6:**
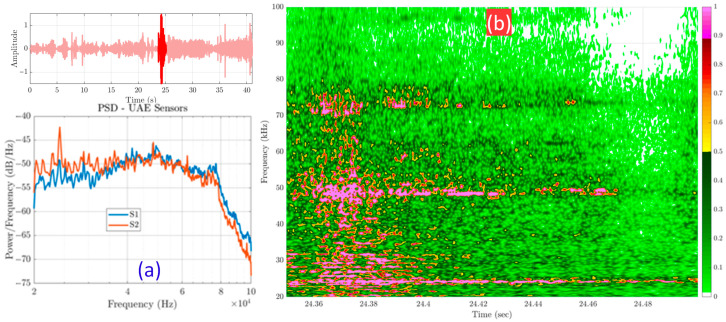
Detailed view of wayside noise. (**a**) A representative high-energy burst and its broadband PSD. (**b**) The corresponding spectrogram, showing a dense and pattern less “wall of sound” across the ultrasonic spectrum.

Figure 5 presents representative data from the field test. The Power Spectral Density (PSD) plots consistently reveal a flat, high-energy, broadband response that saturates the entire ultrasonic band of interest (20–80 kHz). This is precisely the frequency range where crack-induced UAE signatures were expected, leading to a direct and inseparable overlap between the potential target signal and background noise. Further time-frequency analysis (Figure 6) illustrates this phenomenon as a dense, patternless “wall of sound” where the ultrasonic spectrum is saturated with transient energy.

This finding emphasized a fundamental problem for wayside deployment: without a well-defined ground-truth fingerprint of wheel crack emissions, it is nearly impossible to distinguish diagnostic signals from uncontrolled field recordings. Therefore, the outcome of this initial attempt motivated the next phase of the study; specifically, to systematically characterize and validate the true acoustic signature of wheel damage under controlled conditions before returning to the field with improved sensing and processing strategies.

## 4. Experimental Framework for Acoustic Signature Characterization

To address the wayside noise challenge, a comprehensive experimental framework was developed to systematically isolate, define, and validate the ground-truth acoustic signature of wheel damage. The overall experimental setup involved controlled impact testing on two separate collections of railroad wheelsets. The acoustic response was recorded using a multi-sensor arrangement that included both high-frequency ultrasonic (UAE) microphones and audible-range sonic microphones. The resulting data were then processed through a robust, automated analysis pipeline to extract diagnostic features and assess their statistical validity.

### 4.1. Experimental Wheelset Libraries and Test Conditions

Two libraries of wheelsets were used to progressively increase the realism of the test conditions.

**Figure 7:**
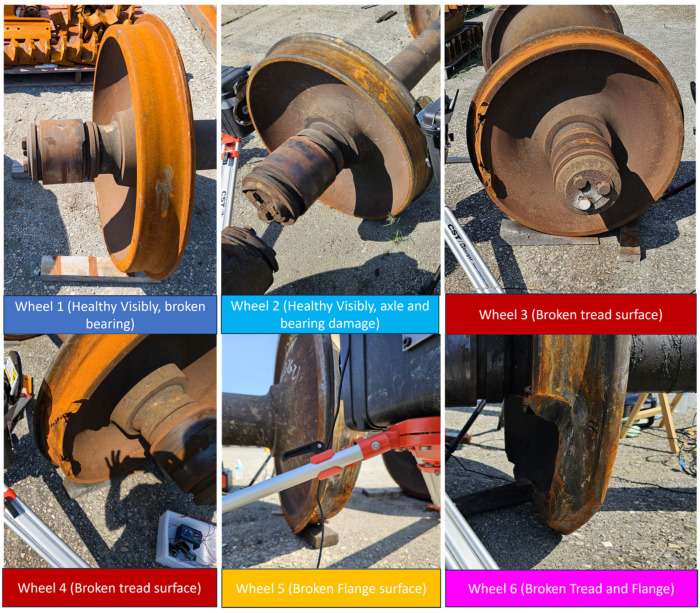
Wheelset library with overt damage conditions as mentioned.

The initial group of wheels included six out-of-service wheelsets with significant structural damage. This group, shown in Figure 7, consisted of two healthy wheels, two with broken rims, one with a broken flange, and another with damage to both the rim and flange. For these tests, each wheelset was supported on wooden blocks to reduce environmental acoustic coupling and establish a clean baseline for initial signature analysis.

**Figure 8:**
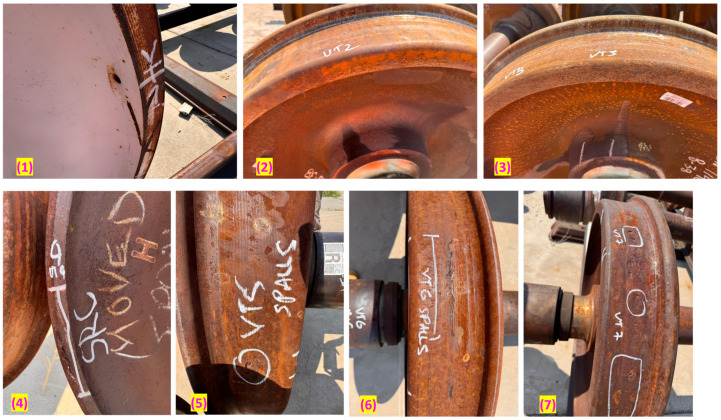
Wheelset library with incipient defects (set 1). (1) Blind holes on rim. [BH] (2,3) Healthy [H]. (4) SRC. (5) Spalling [SP] + SRC + RCF (6) RCF + Spalls [SP] (7) RCF.

**Figure 9:**
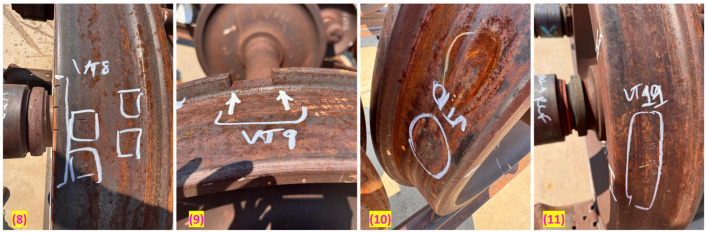
Wheelset library with incipient defects (set 2) (8) Notches [NT] (9) Flange damage [FL] (10) Spall [SP] + notches [NT] (11) RCF.

The second library, shown in Figure 8 and Figure 9, included eleven wheelsets with various common, service-induced or artificially induced incipient defects sourced from the MxV Rail facility. Defect types consisted of Shattered Rim Crack (SRC), Rolling Contact Fatigue (RCF), spalls (SP), blind holes (BH), and notches (NT). Importantly, these tests were performed with the wheelsets resting on sections of rail track. This setup provided realistic physical boundary conditions and acoustic coupling, representing a key step toward simulating in-service conditions and confirming the signature’s robustness against a more challenging baseline.

### 4.2. Instrumentation and Test Protocol

To analyze the damage signature across a broad frequency spectrum, a four-sensor array was employed. The main sensors were two Avisoft Bioacoustics CM24 and CM16 (Avisoft Bioacoustics, Berlin, Germany) ultrasonic microphones (UAE sensors), as described in Section 2.1.

Additionally, the test suite was expanded to include two PCB Piezotronics Model 130D20 sonic microphones (PCB Piezotronics, Inc., Depew, NY, USA) to evaluate the signature’s presence in the audible range and assess the feasibility of a lower-cost sensing alternative. The 130D20 is a prepolarized condenser microphone with an integrated ICP^®^ preamplifier, a sensitivity of 45 mV/Pa, and a frequency response from 20 Hz to 20 kHz.

**Figure 10:**
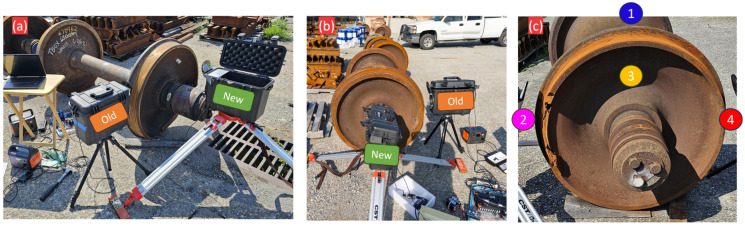
Experimental setup for hammer test data collection. (**a**,**b**) Two UAE sensors (Radial “Old” and Axial “New”) positioned to capture acoustic data. (**c**) The seven distinct locations on the wheel that were systematically excited by a hammer.

Data for all experiments was collected using the Dewesoft SIRIUSi-HD-16xSTGS system at a sampling rate of 200 kHz. Each wheel was systematically excited with a standard hammer at seven different locations, including the top rim/flange (1), wheel plate (3), and side rim/flange areas (2) and (4) (Figure 10c). For each hammer strike, data was recorded simultaneously from all four microphones. The sensors were positioned in both radial (facing the wheel tread) and axial (facing the wheel plate) orientations to capture acoustic energy propagating in different directions, with the axial configuration being more representative of a practical wayside deployment geometry.

### 4.3. Automated Signal Processing and Feature Extraction Pipeline

**Figure 11:**
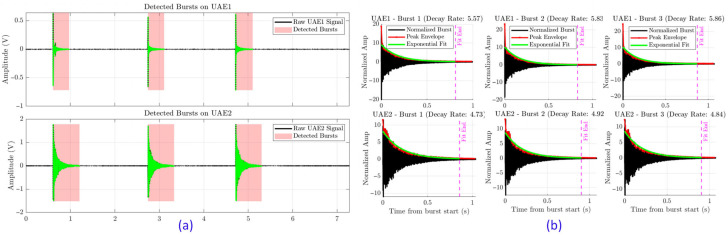
The automated analysis pipeline. (**a**) Automatic detection and isolation of acoustic bursts from the raw signal. (**b**) For each burst, the signal envelope is extracted and fit with an exponential curve to calculate the decay rate.

An automated algorithm was developed to isolate burst events from continuous recordings (Figure 11a). The detector used a dual-threshold approach combining Short-Time Energy (STE) and Line Length (LL) features [23]. This method, adapted from audio and EEG signal processing, filters out background noise while capturing impact-induced bursts.

For each isolated burst, two key time-domain features were computed:

- Decay rate: This feature measures how quickly acoustic energy diminishes. It was determined by first extracting the signal envelope of the burst and then fitting an exponential curve to its decay portion [24] (Figure 11b). The main hypothesis is that internal friction and micro-slip at crack interfaces act as damping mechanisms, resulting in faster dissipation of acoustic energy and a significantly higher decay rate compared to a healthy, resonant wheel.
- RMS Energy: The root-mean-square energy of the burst (in the ultrasonic band 20 to 80 kHz) was calculated to quantify its overall ultrasonic range intensity.

The frequency content of each burst was analyzed to extract features characterizing its spectral properties:

- Spectral Centroid: Calculated as the “center of mass” of the power spectrum, this feature indicates where most of the spectral energy is concentrated [25]. The hypothesis is that damage may change the wheel’s resonant modes, resulting in a shift in the centroid.
- Spectral Entropy: This feature measures the flatness or complexity of the spectrum [26]. A pure tone has low entropy, while white noise has high entropy. It is hypothesized that frictional rubbing at crack faces introduces broadband components, thus altering the spectral entropy.
- Spectral Standard Deviation (Std): Measures the spread of the spectrum around its centroid.
- Band Power Ratio (BPR): The ratio of energy in different frequency bands, specifically $\frac{Energy\;in\;the\;ultrasonic\;range\;(20\;kHz-100\;kHz\;(nyquist)\;)}{Energy\;in\;the\;audible\;sonic\;range\;(1000\;Hz-20\;kHz)}$.

### 4.4. Statistical Validation Framework

To ensure that feature separations between healthy and damaged wheels were statistically significant and not due to random variation, a multi-layered validation framework was used.

1. First, two-sample t-tests were used to assess mean differences, with Levene’s test [27] applied to determine equality of variances. A significance threshold of *p* < 0.05 was adopted, and Bonferroni correction was applied to account for multiple comparisons. For features exhibiting non-normal distributions, Mann–Whitney U tests [28] were conducted to provide non-parametric confirmation of group differences.
2. Beyond significance testing, effect sizes were calculated using Cohen’s d [29], offering a measure of the magnitude of separation. Following established guidelines, d values greater than 0.8 were interpreted as large effects, indicating features with practical diagnostic significance.
3. Finally, Receiver Operating Characteristic (ROC) [30] analysis was employed to evaluate classification performance. The Area Under the Curve (AUC) served as a robust single metric of discriminative ability, where AUC = 1.0 indicates perfect classification and AUC = 0.5 corresponds to random guessing.
4. For AUC, 95% confidence intervals were estimated with a class-stratified bootstrap (2000 resamples). First, the score direction was fixed: AUC was computed once with the raw feature and once with its negative; the larger value set the direction and was used for all resamples. In each resample, healthy and damaged cases were drawn within their own groups (with replacement) to keep the class balance, and a new AUC was computed. The 2.5th and 97.5th percentiles of these 2000 AUC values formed the 95% CI. A fixed random seed makes the result repeatable. CIs are omitted if either class has fewer than two samples.

This statistical framework confirmed that the results were genuine (using *p*-values and non-parametric tests), quantified the magnitude of separation (using effect sizes), and provided uncertainty bounds on discriminative performance (bootstrap 95% CIs for AUC). In this way, only the features that were both reliable and distinctly different between healthy and damaged wheels were retained as strong candidates for classification.

## 5. Results: Characterization and Validation of a Damage-Induced Acoustic Fingerprint

This section presents the empirical findings from the experimental framework. The results are presented in a logical progression, beginning with the initial discovery of the acoustic fingerprint using UAE sensors on overtly damaged wheels, followed by a comparative analysis across ultrasonic and sonic frequency bands, and finally ending in the validation of the signature on a challenging group of wheels with service-induced incipient defects.

### 5.1. Discovery of a Robust Damage Fingerprint with UAE Sensors

The initial investigation of wheelsets that included wheels with obvious, large-scale structural damage revealed a highly robust and statistically significant acoustic fingerprint. Data from both raw signals and band-pass filtered signals (20–80 kHz) were analyzed to specifically isolate and assess the ultrasonic content, as frequencies above 80 kHz approach the 100 kHz Nyquist limit and contain less information.

**Figure 12:**
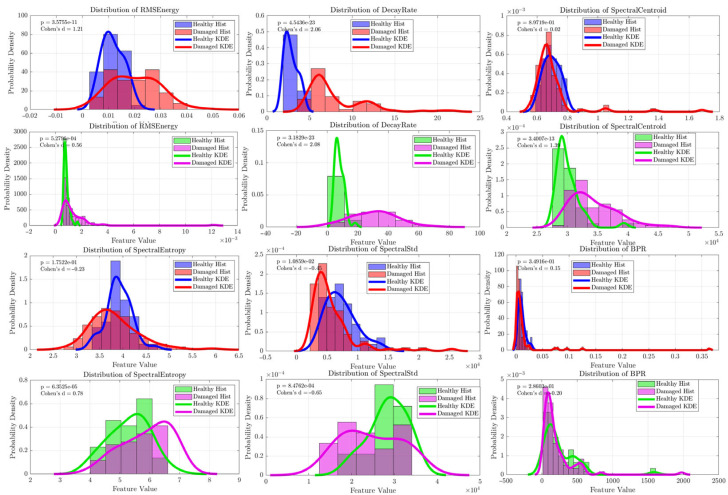
Kernel density distributions of key features for the Radial sensor. Healthy wheels are shown in blue (raw data) and green (band-pass filtered, 20–80 kHz), while damaged wheels are shown in red (raw data) and magenta (band-pass filtered).

**Figure 13:**
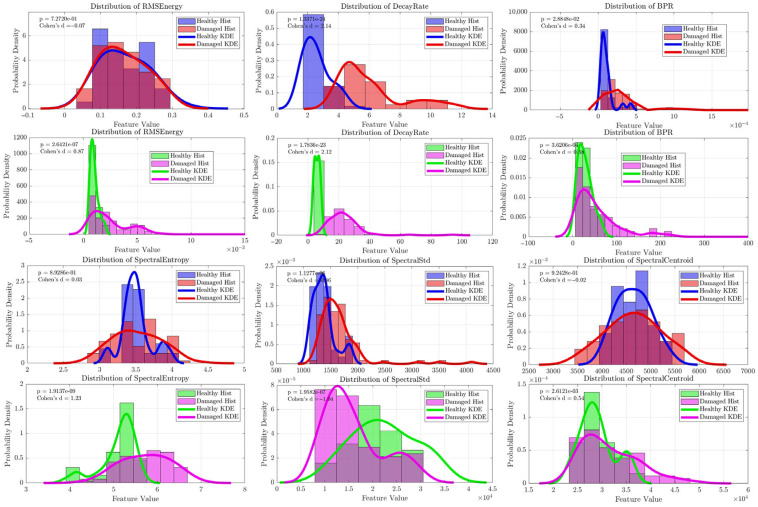
Kernel density distributions of key features for the Axial sensor. Healthy wheels are shown in blue (raw data) and green (band-pass filtered, 20–80 kHz), while damaged wheels are shown in red (raw data) and magenta (band-pass filtered).

**Figure 14:**
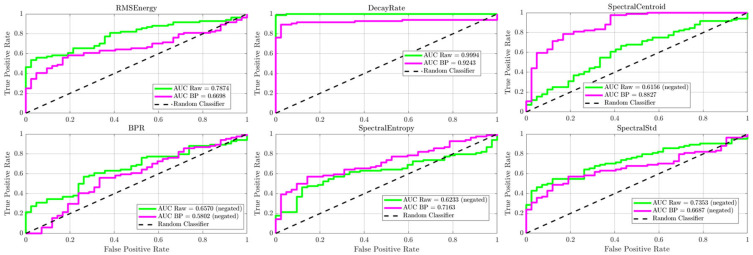
ROC analysis for the Radial sensor. The near-perfect AUC (Area under curve) for decay rate confirms its status as a premier diagnostic classifier.

**Figure 15:**
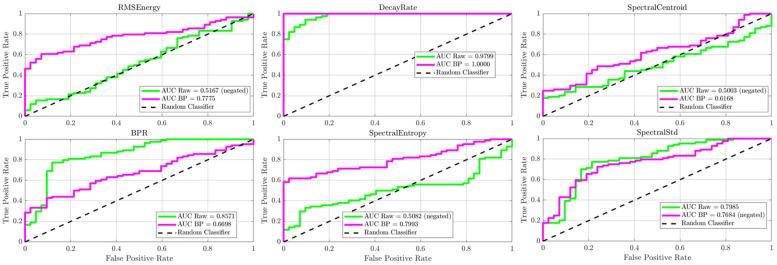
ROC analysis for the Axial sensor, validating decay rate as a perfect classifier and demonstrating the strong diagnostic power of other spectral features.

The most important finding from this phase was the emergence of the time-domain **decay rate** feature as a leading diagnostic classifier. As shown by the Kernel Density Estimation (KDE) plots in Figure 12 and Figure 13, the decay rate demonstrated a clear separation between the healthy and damaged wheel populations, a separation that was visibly improved by the band-pass filtering process. For both the radial and axial sensor orientations, this feature achieved nearly perfect classification performance, with Area Under the Curve (AUC) values consistently exceeding 0.98 and even reaching a perfect 1.0 for the filtered axial sensor data (Figure 14 and Figure 15). With highly significant *p*-values (*p* << 0.001), this result identified acoustic damping within the ultrasonic band as a primary and highly reliable indicator of overt wheel damage. For the decay-rate ROC specifically, the bootstrap 95% CIs were: band-pass axial [1.00, 1.00], band-pass radial [0.8651, 0.9715], raw axial [0.9580, 0.9946], and raw radial [0.9972, 1.00].

Although decay rate provided the clearest separation, several frequency-domain features also showed diagnostic value, though to a lesser extent. Features such as Spectral Std and Spectral Entropy exhibited statistically significant differences between the classes, as shown by their distinct KDE plots (Figure 12 and Figure 13). Notably, the classification accuracy of these spectral features, especially Spectral Entropy, improved markedly after band-pass filtering, with AUC values rising from approximately 0.6 to 0.7 for the radial sensor (Figure 14). These findings suggest that wheel damage causes measurable changes in the spectral content of the acoustic response and confirm that analyzing the 20–80 kHz ultrasonic band effectively eliminates low-frequency structural noise, thus strengthening the diagnostic capability of the acoustic signature.

### 5.2. Comparative Analysis of Ultrasonic and Sonic Signatures

To assess the broadband nature of the discovered signature and evaluate a lower-cost sensing alternative, a comparative analysis was conducted between ultrasonic UAE sensors and audible-range sonic sensors.

Based on the findings from Section 5.1, where filtering was shown to improve feature separation, all subsequent analyses were conducted on filtered data. For the UAE sensors, a band-pass filter of 20 to 80 kHz was applied to isolate the ultrasonic signature. For the new sonic sensor, a bandpass filter of 1000 Hz to 20 kHz was used to focus on the relevant audible spectrum while eliminating low-frequency mechanical noise and high-frequency aliasing. To clarify, the following analysis only presents the most diagnostically relevant features that demonstrated clear separation between healthy and damaged wheels.

**Figure 16:**
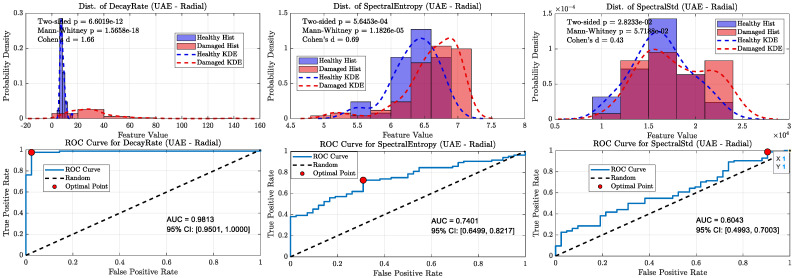
Kernel density distributions and ROC curves for key features using the UAE radial sensor (20–80 kHz band-pass).

**Figure 17:**
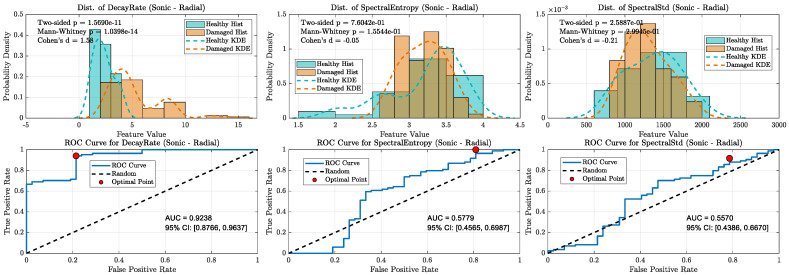
Kernel density distributions and ROC curves for key features using the Sonic radial sensor (1-20 kHz band-pass).

**Figure 18:**
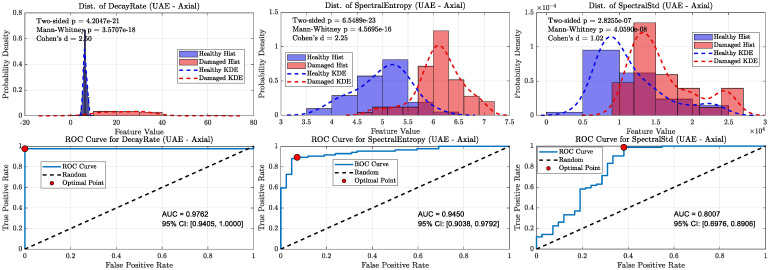
Kernel density distributions and ROC curves for key features using the UAE axial sensor (20–80 kHz band-pass).

**Figure 19:**
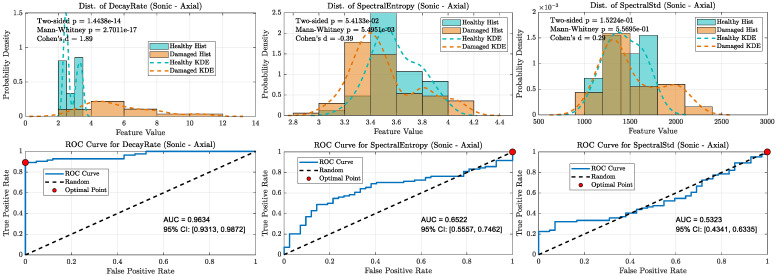
Kernel density distributions and ROC curves for key features using the Sonic axial sensor (1–20 kHz band-pass).

A key finding from this analysis, shown in Figure 16, Figure 17, Figure 18 and Figure 19, is the notable robustness of the decay rate feature across different frequency bands. Even when measured with sonic sensors and filtered to the 1–20 kHz band, the decay rate still showed strong classification performance. However, a direct comparison reveals the superior fidelity of the ultrasonic band: while the sonic sensors achieved excellent AUC values of approximately 0.92 to 0.96 for decay rate, the UAE sensors achieved near-perfect AUCs of approximately 0.98. This suggests that, although the damage-induced damping effect is a broadband phenomenon visible in both regimes, ultrasonic measurement offers greater confidence for classification. As demonstrated in the following section, this performance difference increases when analyzing more subtle or incipient defects.

In contrast, the diagnostic ability of the spectral features significantly decreased in the sonic band, with their respective AUC values dropping to near-random levels (~0.55–0.65). This highlights the importance of the ultrasonic frequency range for extracting detailed spectral data and confirms that while sonic sensors can detect the dominant damping effect, UAE sensors are better suited for a more comprehensive, multi-feature analysis.

### 5.3. Performance Validation on Service-Induced Incipient Defects

The final phase of the investigation was to verify the acoustic fingerprint’s effectiveness on wheelsets with early-stage, service-related incipient defects, which are much smaller than the wheels with significant damage discussed earlier. The wheelsets were placed on a rail to simulate a service-like boundary condition between the wheel and rail.

The tests introduced two critical elements that greatly enhanced their relevance to the UAE sensors implementation in practice. First, all wheelsets were mounted on tracks, providing a more accurate boundary condition compared to what a rolling wheel experiences. Second, the wheelsets included wheels with a variety of common, service-induced defects (Section 4.1, Figure 8 and Figure 9) that are often difficult to detect or not visible, including:

1. Rolling Contact Fatigue (RCF)
2. Shattered Rims (SRC)
3. Spalls (SP)
4. Blind Holes (BH)
5. Notches (NT)
6. Flange Damage (FL)

The same systematic hammer test protocol was used, exciting the wheels at specific locations and recording the acoustic response with both Radial and Axial UAE sensors.

**Figure 20:**
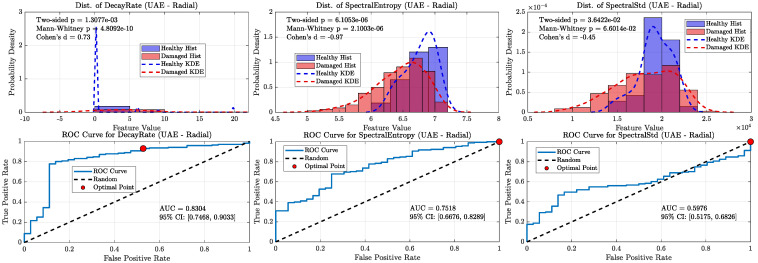
Statistical distributions and ROC curves for UAE Radial sensor features (decay rate, Spectral Entropy, Spectral Std) comparing healthy and damaged wheel populations.

**Figure 21:**
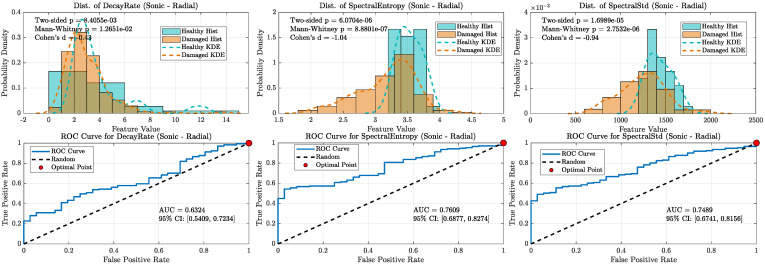
KDE and ROC curves for the Sonic radial sensor (1–20 kHz). decay rate performance drops substantially (AUC ≈ 0.63), confirming limited suitability of sonic sensing for incipient defect detection.

**Figure 22:**
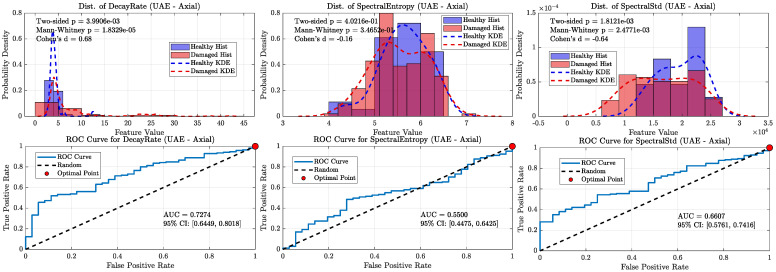
Statistical distributions and ROC curves for UAE Axial sensor features, highlighting decay rate as the strongest classifier, with moderate contributions from spectral features.

**Figure 23:**
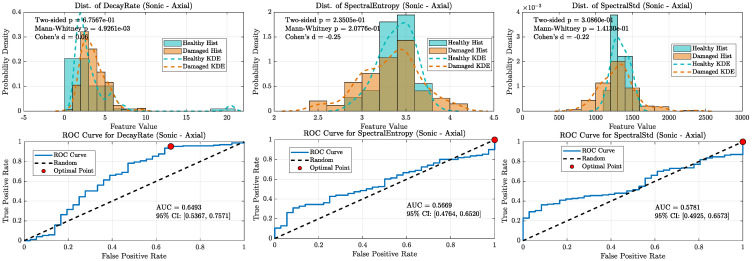
KDE and ROC curves for the Sonic axial sensor (1–20 kHz). Classification power of decay rate further degrades (AUC ≈ 0.66), and spectral features approach random levels, highlighting the weakness of sonic sensors in this regime.

When all damaged wheels in this library were grouped together, the overall classification performance became more nuanced, clearly distinguishing the capabilities of the ultrasonic and sonic sensors (refer Figure 20, Figure 21, Figure 22 and Figure 23). As shown in the aggregate KDE and ROC plots for the UAE sensors (Figure 20 and Figure 22), the decay rate feature still provided a statistically significant separation between the healthy and damaged classes, achieving an AUC of 0.8266 for the radial sensor and 0.7344 for the axial sensor. While lower than the near-perfect separation seen with overt damage, this result confirms that the UAE signature remains a valid indicator even for more subtle defects.

In stark contrast, the performance of the sonic sensors declined significantly on this challenging dataset. As shown in Figure 21 and Figure 23, the decay rate feature measured by the sonic sensors exhibited much weaker separation, with AUC values dropping to 0.6296 (radial) and 0.6610 (axial). This clear divergence confirms that while sonic sensors can detect the strong damping effect of overt damage, they lack the sensitivity needed to reliably identify the more subtle signatures of incipient defects. The spectral features for both sensor types showed near-random classification performance, with all AUCs falling below 0.78, further supporting this conclusion.

**Figure 24:**
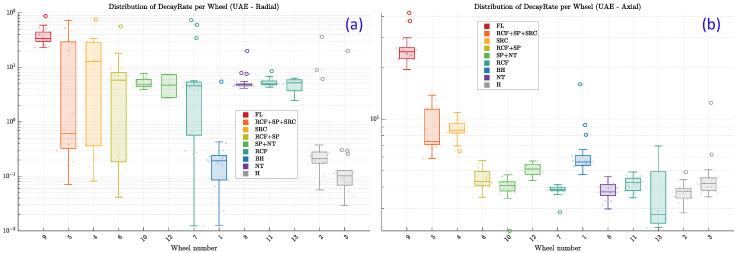
Box-plot distributions of Decay Rate per wheel for (**a**) the Radial UAE sensor and (**b**) the Axial UAE sensor. Individual data points are overlaid. Wheels are grouped by defect type (legend) and ordered left-to-right by decreasing visually assessed damage severity to aid interpretation (ordering used only for visualization). The y-axis is logarithmic to show spread across orders of magnitude.

Given the superior performance of the UAE sensors, a more detailed, defect-specific analysis was conducted using only the UAE data to better understand the underlying physics. To visualize the distribution and statistical spread of the decay rate for each wheel, box plots are presented in Figure 24. For clarity of presentation, wheels are displayed left-to-right by decreasing visually assessed damage severity (subjective ordering used only for visualization), which reveals a clear trend-most evident for the axial orientation in Figure 24b. This analysis offers valuable insights that directly support and refine the core physical hypothesis of this study. The results revealed two distinct patterns:

- High Sensitivity and Clear Separation for Frictional/Geometric Defects: The box plots for wheels with defects known to involve internal frictional interfaces, such as Shattered Rim Crack (SRC, seen in Wheels 4 and 5), or significant geometric changes and mass loss, like Flange Damage (FL, Wheel 9), show a clear and statistically significant shift to higher Decay Rate values. Notably, the interquartile ranges (the boxes) for these defect types show minimal to no overlap with the distributions of the healthy wheels (H), indicating a robust separation (especially for the axial sensor orientation). With the severity-ordered display, the median Decay Rate increases with apparent damage severity, reinforcing the expected damping trend, particularly in the axial sensor. The higher median values and wider data spread for these wheels strongly corroborate the hypothesis that the method is highly sensitive to conditions that either increase frictional damping or fundamentally alter the wheel’s natural modes of vibration.
- Overlapping Distributions for Non-Frictional Defects: Conversely, the box plots for wheels with surface or near-surface defects that do not inherently create frictional interfaces, such as Rolling Contact Fatigue (RCF) and spalls (SP), show distributions that are largely indistinguishable from those of the healthy wheels (Figure 24b). Their median Decay Rates are similar, and their interquartile ranges exhibit significant overlap. This key finding, clearly visualized by the box plots, reveals the current limitations of relying on a single feature for classification. It highlights that under this controlled hammer-impact test configuration, these types of defects do not produce a unique damping signature and will likely require more advanced, multi-feature classification models for reliable detection.

## 6. Paths Forward: From Fingerprint to In-Motion Deployment

### 6.1. Considerations for Robustness in Wayside Deployment

A key consideration for wayside deployment is the effect of operational variables such as train speed and adverse weather. However, the leading diagnostic feature identified in this work—the exponential decay rate of burst envelope, is a fundamental damping property of the wheel/defect system. As it is computed in the time domain, it is largely invariant to train speed. Higher speeds might change the burst occurrence rate and modestly shift spectral content via a small Doppler factor (v ≪ c), but this does not alter the decay rate of an individual burst once an impact-like event occurs. Weather primarily affects the air-path signal-to-noise ratio (through wind and rain noise) and frequency-dependent attenuation (due to temperature and humidity). These effects can be addressed via physical directivity (waveguides), environmental logging, and lightweight corrections to the power spectral density before feature calculation. Crucially, none of these mechanisms invalidate the fundamental separation provided by the decay-rate feature; they affect its detectability. Therefore, these challenges are treated as an SNR engineering problem rather than a limitation of the classifier itself.

### 6.2. The Remaining Challenge: Isolating Wheel-Specific Signals

**Figure 25:**
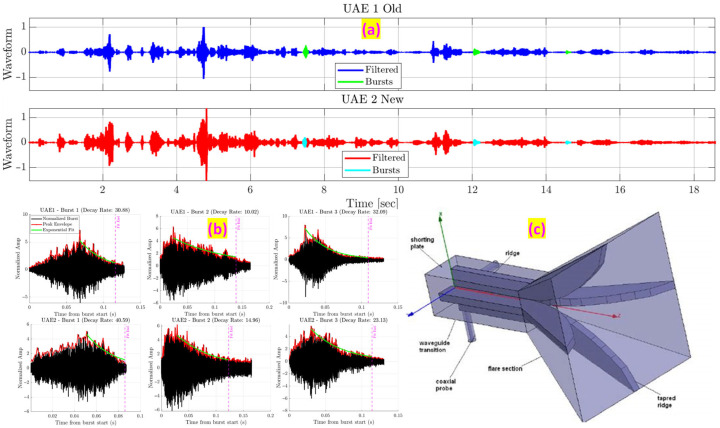
(**a**) Application of the burst detection algorithm to wayside data successfully isolates impact-like events. (**b**) The feature extraction pipeline applied to these real-world bursts. (**c**) A conceptual design for a passive acoustic waveguide to address the challenge of off-axis noise.

Although the systematic hammer tests offered a controlled method for capturing a validated acoustic fingerprint of wheel damage, applying these results to in-motion conditions remain difficult. As shown in Figure 25a,b, impact-like bursts also show up in wayside recordings. These bursts occur during wheel-rail contact under load and generate high-energy ultrasonic transients analogous to those recorded in controlled hammer tests.

However, the main challenge is ensuring that detected bursts come from the wheel being inspected rather than external, off-axis noise sources. Field tests showed that the current microphones, with a wide aperture of about 100°, capture substantial interference from surrounding components such as draft gears, suspensions, and other mechanical systems. This interference occurs in the same frequency range (20–80 kHz) as the ultrasonic signals of interest, making digital filtering alone ineffective.

### 6.3. Proposed Solution: Physical Acoustic Focusing with Waveguides and Development of an Automated Diagnostic Model

To address the issue of off-axis noise, our approach goes beyond digital signal processing and involves a physical hardware solution: passive acoustic waveguides. As shown conceptually in Figure 25c, a waveguide functions as a “sound lens” or an inverted acoustic horn, designed to improve the sensor’s directionality.

The mechanism works by narrowing the sensor’s “listening cone.” The waveguide is designed to efficiently capture sound waves originating from a specific target area, such as the wheel’s rim and flange interacting with the rail, and to direct them directly to the microphone’s diaphragm. At the same time, it physically blocks or reduces sounds emanating from off-angle directions. By physically focusing the sensor’s sensitivity on the target, we aim to significantly boost the signal-to-noise ratio (SNR) of the wheel-specific acoustic events, making our validated acoustic fingerprint much easier to detect amid interfering trackside noises.

While improving SNR is the main focus, the ultimate aim is to develop a fully automated diagnostic system. The rich, multi-sensor feature library created during the ground truth and advanced validation phases offers an excellent foundation for building a sophisticated machine learning classifier. We plan to train and evaluate various models (e.g., Support Vector Machines (SVM), Decision Trees, Neural Networks) using our existing dataset. The goal is to develop a robust model that can take features from an acquired acoustic burst as input and produce a reliable classification of the wheel’s health status. This data-driven method will learn the complex non-linear relationships among multiple features, potentially enabling the detection of more subtle defect types, such as RCF, which were difficult to identify with a single feature alone.

## 7. Conclusions and Future Work

### 7.1. Conclusions

This study successfully evaluated the use of passive air-coupled ultrasonic acoustic (UAE) sensors for detecting railroad wheel damage. Through a series of tests in various conditions, it addressed the fundamental challenge of defining the acoustic signature of railroad wheel damage, an essential step for creating a passive, in-motion wayside monitoring system. The tests resulted in several key conclusions:

1. A robust and statistically validated “acoustic fingerprint” for railroad wheel damage can be identified and confirmed. The most effective and dependable feature within this fingerprint is the acoustic decay rate, which assesses how quickly the signal diminishes. This feature achieved near-perfect classification (AUC > 0.95) between healthy wheels and those with obvious structural damage.
2. The diagnostic signature is directly connected to the fundamental physics of the defect. The method’s high sensitivity to defects that involve internal frictional interfaces (for example, shattered rim cracks) and major geometric changes (such as flange damage) supports the core physical hypothesis of the passive UAE method: that frictional cracks and large geometric changes speed up energy dissipation and can be detected through broadband ultrasonic emissions.
3. The ultrasonic frequency band (20–80 kHz) provides the highest-quality diagnostic information. Although the main decay rate signature is a broadband phenomenon detectable by sonic sensors, the ultrasonic band offers superior classification confidence and contains richer spectral data needed for a more comprehensive, multi-feature analysis.

Ultimately, this research provides the validated ground-truth signature that was previously missing. With this foundation, the challenge of detecting defects in motion is no longer a blind search but a focused engineering problem. This knowledge is the crucial step toward developing a practical, affordable, and non-contact system for monitoring wheel health.

### 7.2. Future Work

To achieve the ultimate goal of revenue-service deployment, the following staged roadmap is proposed, building directly on the validated acoustic fingerprint:

1. Laboratory Validation and Hardware Refinement

The first step is to design and implement passive acoustic waveguides that function as a “sound lens,” directing sensor sensitivity towards the wheel while attenuating off-axis noise. As conceptualized in Figure 25c and Appendix A, these waveguides will address the “wall of sound” challenge by improving signal-to-noise ratios without relying on active components.

2. Advanced Classification Models

In parallel to designing waveguide, we will also focus on developing multi-feature machine learning classifiers, such as support vector machines (SVMs) and neural networks, trained on the comprehensive feature library (e.g., decay rate fused with spectral metrics). This will improve sensitivity to subtle, non-frictional defects such as RCF and spalls, and allow the system to move beyond the limitations of single-feature detection.

3. Integrated Field Demonstration

The final milestone is to integrate these hardware (waveguide-enhanced sensors) and software (ML classifiers) solutions into a complete prototype system. Deploying this system in an in-motion revenue-service environment will provide a definitive demonstration of feasibility and establish the technology’s readiness level for practical railway safety and maintenance applications.

## Data Availability

The data presented in this study are available on request from the corresponding author. The data are not publicly available due to ongoing research and intellectual property considerations related to the funded project.

## Abbreviations

AAR	Association of American Railroads
AE	Acoustic Emission
AUC	Area Under the (ROC) Curve
BH	Blind Hole(s) (defect label)
BPR	Band Power Ratio
DAQ	Data Acquisition system
EMAT	Electromagnetic Acoustic Transducer
EEG	Electroencephalography
FL	Flange Damage (defect label)
FRA	Federal Railroad Administration
H	Healthy (class label)
ICP^®^	Integrated Circuit Piezoelectric (constant-current sensor interface)
KDE	Kernel Density Estimation
LL	Line Length (burst detection feature)
NT	Notch/Notches (defect label)
PSD	Power Spectral Density
RCF	Rolling Contact Fatigue
ROC	Receiver Operating Characteristic
RMS	Root-Mean-Square
SNR	Signal-to-Noise Ratio
SP	Spalls (defect label)
SRC	Shattered Rim Crack
STD	Standard Deviation
STE	Short-Time Energy (burst detection feature)
SVM	Support Vector Machine
UAE	Ultrasonic Acoustic Emission
UT	Ultrasonic Testing
VSR	Vertical Split Rim
CI	Confidence Interval

## Appendix A. Preliminary Design Calculations for Cylindrical Waveguide

### Appendix A.1. Governing Relations

The effective acceptance half-angle of a waveguide coupled to the CM24 microphone is set by the most restrictive of three limits [31]:

$$\theta_{eff}\left(f\right)=\min\left(\theta_{sensor},\;\theta_{geo}=\tan^{-1}\frac{\frac{D}{2}}{L_{eff}},\;\theta_{diff}\left(f\right)=0.886\frac{\lambda }{D}\right)$$

where:

- D = 44 mm: sensor diaphragm/throat diameter (fixed),
- $L_{eff}$: diaphragm-to-mouth effective length,
- $\theta_{sensor}$ = 20°: CM24 polar response (±20° total aperture),
- c ≈ 343 m/s

At stand-off R = 1.27 m, the accepted footprint along the rail is:

$$w\left(f\right)=2Rtan\;\theta_{eff}(f)$$

and the burst time window at train speed v is:

$$T\left(f,\;v\right)=\frac{w(f)}{v}$$

To ensure capture of the full ring-down envelope from even the slowest-decaying (“healthy”) wheels, the time window must exceed three time constants $\left(3\tau \right)$:

$$T\left(f,\;v\right)\geq 3\tau \;,\;\tau =\frac{1}{\alpha },\;\alpha \;\left(decay\;rate\right)\leq 4s^{-1}$$

Thus, T ≥ 0.75 s is the conservative requirement for a healthy wheel.

### Appendix A.2. Fixed Inputs

- Sensor throat/mouth: D = 44 mm
- Stand-off: R = 1.27 m
- Sensor polar: ±20° (total 40°).
- Frequency range of interest: 20-80 kHz.
- Train speed range (initial validation): 20-40 mph (8.9–17.9 m/s).

### Appendix A.3. Selection Chart

The table below shows the effective acceptance half-angle, footprint width, and time windows for several geometry caps (achieved by setting $L_{eff}$):

**Table A1:** Effective Acceptance Angles, Footprints, and Time Windows for Candidate Cylindrical Waveguide Geometries.

$\mathrm{Geometry}\;\mathrm{Cap}\;(\theta_{geo})$	$L_{eff}\;(mm)$	f (kHz)	$\theta_{eff}$ Used (deg at 20/40/80 kHz)	Footprint $w\left(f\right)$ @ R = 1.27 m (cm)	T @ 20 mph (ms)	T @ 30 mph (ms)	T @ 40 mph (ms)
20°	~60	20-80	~20	~92–95	185–190	123–127	92–95
12°	~105	20–80	12/8.7/4.3	53/39/19	106/78/38	71/52/25	53/39/19
10°	~125	20–80	10/8.7/4.3	45/39/19	91/78/38	61/52/25	45/39/19
8°	~157	20–80	8/8.7/4.3	36/39/19	73/78/38	49/52/25	36/39/19

All configurations produce T ≫ 0.75 s, thus comfortably capturing at least 3τ of the decay envelope even at the highest validation speeds.

### Appendix A.4. Recommended Geometry

A cylindrical guide of **D = 44 mm, L ≈ 150–160 mm**, with the microphone recessed by 10–15 mm ($L_{eff}$ ≈ 135–150 mm), yields an effective half-angle of ~8–9°. This configuration:

- Narrows acceptance compared to the bare sensor polar (improving SNR),
- Preserves sufficient time window for decay-rate analysis,
- Is compact, rugged, and practical for trackside deployment.

Wall damping and a thin hydrophobic membrane at the mouth are also advised to attenuate grazing off-axis noise while protecting the sensor.
